# Supplementary material for: Muropeptides Stimulate Growth Resumption from Stationary Phase in Escherichia coli
Source: Sci Rep. 2019 Dec 2;9:18043. doi: 10.1038/s41598-019-54646-5 (PMC6888817; doi:10.1038/s41598-019-54646-5)
Supplement: Supplementary file 1 — Supplementary information [file 41598_2019_54646_MOESM1_ESM.pdf]

Supplementary Information

Muropeptides Stimulate Growth Resumption from Stationary Phase in *Escherichia coli*

Arvi Jõers, Kristiina Vind, Sara B. Hernández, Regina Maruste, Marta Pereira, Age Brauer, Maido Remm, Felipe Cava, Tanel Tenson

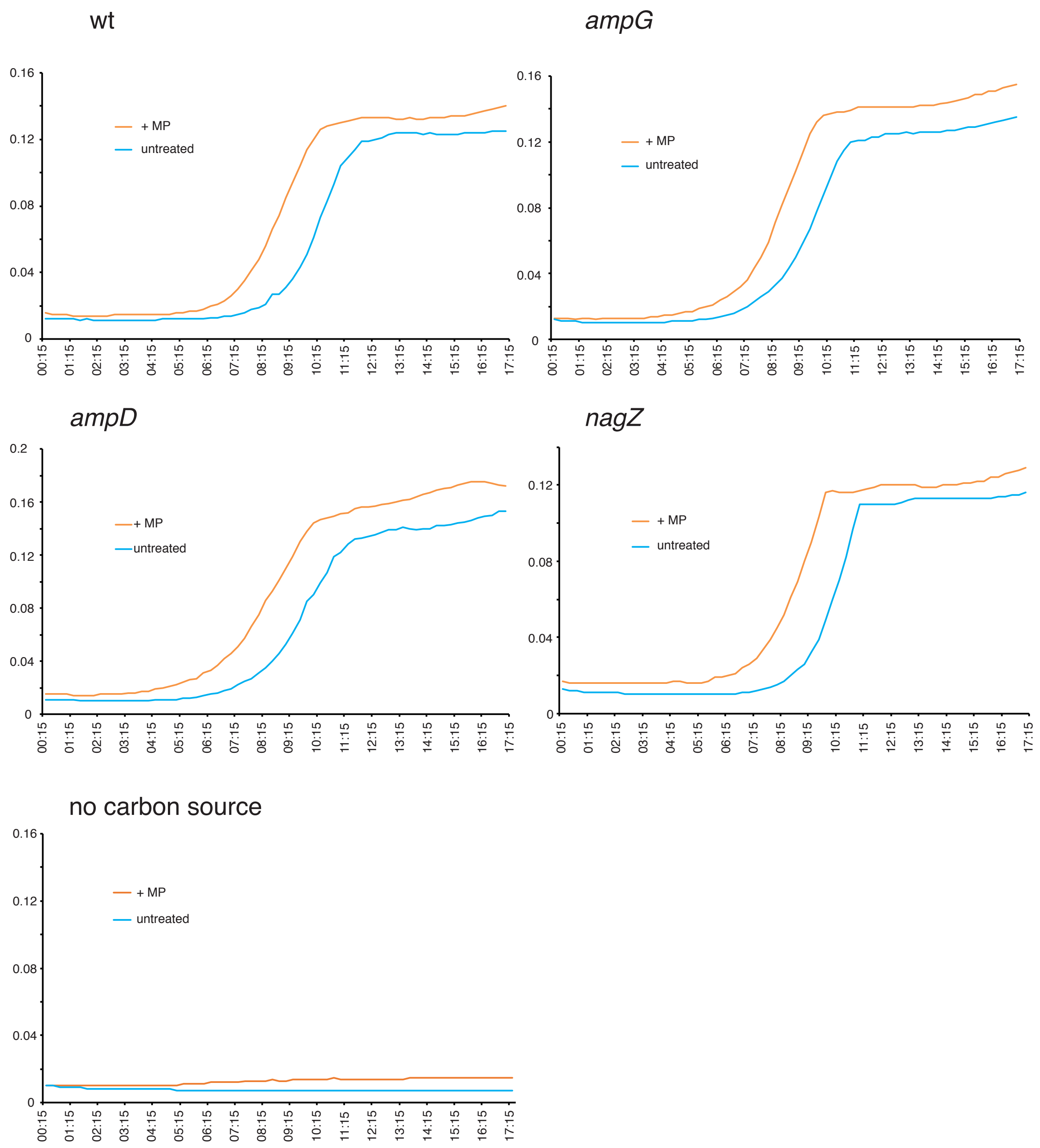

Supplementary Figure S1. Mutants of the muropeptide recycling pathway still respond to muropeptides. MP (80 ng/µl) was added to the recovering cells and the cell growth was monitored by OD. In the bottom left panel wt cells were inoculated to MOPS medium without a carbon source.

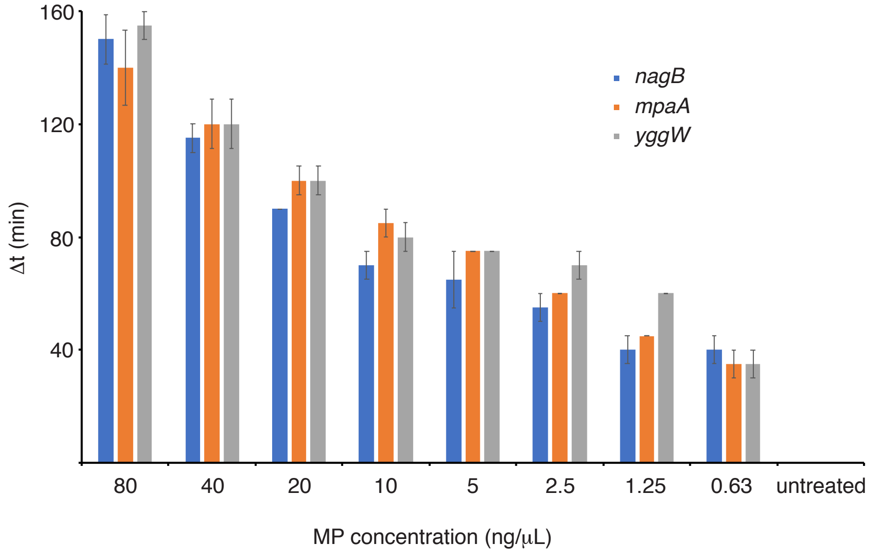

Supplementary Figure S2. Muropeptides stimulate growth resumption of *nagB*, *mpaA* and *yggW* strains. Different amounts of MP or PG were added to recovering cells and the Δt was calculated. The average and standard error of the mean of three independent experiments are shown.

a

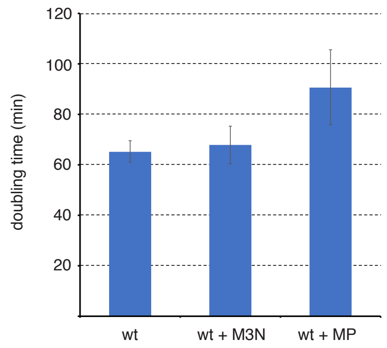

b

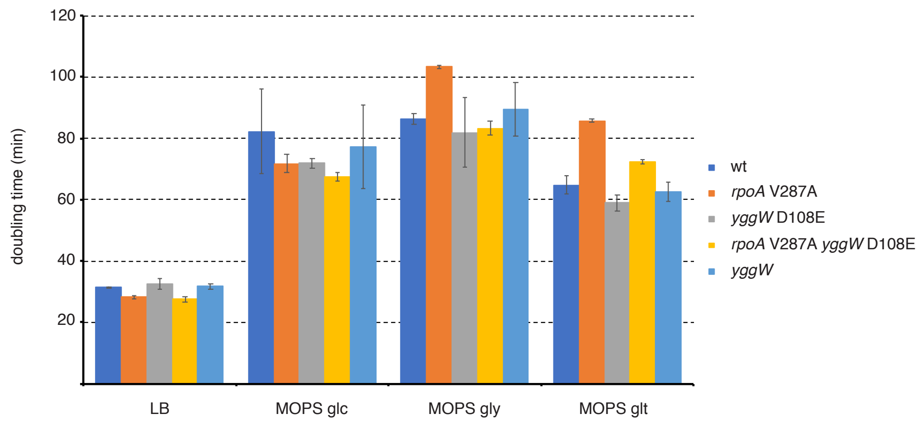

Supplementary Figure S3. Doubling times of different E. coli strains. a. Doubling time of wt (BW25113) strain in the absence and presence of MP. **b.** Doubling times of strains carrying different point mutations in different media. The average and standard error of the mean of three independent experiments are shown.
